# Supplementary material for: Despotism promotes dyadic cooperation through enhanced interdependencies in non-human primate societies
Source: Nat Commun. 2026 Apr 30;17:3513. doi: 10.1038/s41467-026-71168-7 (PMC13133276; doi:10.1038/s41467-026-71168-7)
Supplement: Supplementary file 1 — Supplementary Information [file 41467_2026_71168_MOESM1_ESM.pdf]

Supplementary Information to

Despotism promotes dyadic cooperation through enhanced interdependencies in non-human primate societies

Debottam Bhattacharjee, Tonko W. Zijlstra, Tom S. Roth, Elena Belli, Sophie Calis, Paula Escriche Chova, Eythan Cousin, Jolanda A. de Jong, Edwin J.A.M. de Laat, Aníta Rut Guðjónsdóttir, Karline R.L. Janmaat, Elja J. Jeunink, Charlotte E. Kluiver, Penny E.N. Kuijer, Esmee Middelburg, Lena S. Pflüger, Veera I. Schroderus, Eva S.J. van Dijk, Jonas Verspeek, Sophie Waasdorp, Adam N. Zeeman, Elisabeth H.M. Sterck, Edwin J.C. van Leeuwen, and Jorg J.M. Massen

Corresponding authors:
Debottam Bhattacharjee: bhattacharjee.debottam@gmail.com, Edwin J.C. van Leeuwen: edwin_van_leeuwen@eva.mpg.de, Jorg J.M. Massen: j.j.m.massen@uu.nl

Table S1. Macaque study group details and different tests performed.

Table S2. Details of macaque study groups excluded from partial Bayesian correlation analyses.

Table S3. Effects of dyadic tolerance, rank difference, tolerance grades, prosociality, and kinship on the likelihood of cooperation.

Table S4. Effects of dyadic tolerance, rank difference, tolerance grades, prosociality, and kinship on the magnitude of cooperation.

Table S5. Effects of tolerance grades, group size, groom index, and aggression index on the likelihood of cooperation.

Table S6. Effects of tolerance grades, group size, groom index, and aggression index on the magnitude of cooperation.

Table S7. Effects of dyadic tolerance, rank difference, tolerance grades, and kinship on the likelihood of prosocial food provisioning.

Table S8. Effects of dyadic tolerance, rank difference, and kinship on the magnitude of prosocial food provisioning.

Fig. S1. Dyadic predictors of prosocial food provisioning.

Fig. S2. EMO-model simulation with easy-going LIKE dynamics and fast increase speed shows the emergence of LIKE relationships in societies along a despotic-egalitarian gradient.

Fig. S3. EMO-model simulation with easy-going LIKE dynamics and intermediate increase speed shows the emergence of LIKE relationships in societies along a despotic-egalitarian gradient.

Fig. S4. EMO-model simulation with easy-going LIKE dynamics and slow increase speed shows the emergence of LIKE relationships in societies along a despotic-egalitarian gradient.

Fig. S5. EMO-model simulation with picky LIKE dynamics and fast increase speed shows the emergence of LIKE relationships in societies along a despotic-egalitarian gradient.

Fig. S6. EMO-model simulation with picky LIKE dynamics and slow increase speed shows the emergence of LIKE relationships in societies along a despotic-egalitarian gradient.

References

Table S1. Macaque study group details and different tests performed. Colored cells indicate observations or tests performed, and blank cells indicate that observations or tests could not be performed due to COVID-19 restrictions. N = Sample size, F = Female, M = Male, J = Juvenile and sub-adults (>1 year but ≤ 3.5 years at the time of testing), A = Adults (>3.5 years). In the observations column, total observation minutes are given along with information on mean ± standard deviation per individual in parentheses. For cooperation, the numbers indicate the self-trained participating individuals (cf. **Data S1**). References (#) show the use of published datasets for these tests using experimental designs identical to the current study.

Macaque Species	Group Name	Observations and Tests			
		**Observations**	**Cooperation**	**Prosociality**	**Co-feeding**
Macaca fuscata (Japanese)	Affenberg		F=13, M=3, J=9 , A=7	F=19, M=6, J=9, A=16; # [1]	
Macaca mulatta (Rhesus)	R3G2	N=15; 1300 min (86.66±14.47) min	F=6, M=1, J=3, A=4		N=15, F=13, M=2, J=3, A=12
	R3G7	N=14, 2947.4 min (210.53±18.13) min	F=2, J=2	F=13, M=1, J=4, A=10	N=13, F=12, M=1, J=3, A=10
Macaca fascicularis (Long-tailed)	J1G4	N=11; 3520 min (320) min	F=5, M=2, J=2, A=5; # [2]	F=9, M=2, J=4, A=7; # [3]	N=11, F=9, M=2, J=3, A=8
	J1G7	N=17; 5440 min (320) min	F=6, M=4, J=5, A=5; # [2]	F=13, M=2, J=6, A=9; # [3]	N=17, F=12, M=5, J=7, A=10
	NWR	N=4; 1280 min (320) min	M=4, A=4; # [2]	M=4, A=4; # [3]	N=4, M=4, A=4
Macaca silenus (Lion-tailed)	Blijdorp	N=3; 740 min (246.66±30.55) min	F=1, M=1, A=2	F=3, M=1, A=4	N=3, F=2, M=1, A=3
	Apenheul	N=8; 2820 min (352.5±10.35) min			N=8, F=5, M=3, J=2, A=6
Macaca sylvanus (Barbary)	Gaia	N=14; 3410.7 min (243.62±1.65) min	F=1, M=4, A=5	F=8, M=6, A=14	N=14, F=8, M=6, A=14
	Apenheul	N=9; 3855 min (428.33±7.81) min			N=9, F=8, M=1, A=9
Macaca nigra (Crested)	Blijdorp	N=5; 2353.7 min (470.75±114.44) min	F=1, M=3, J=2, A=2	M=3, F=2, J=2, A=3	N=6, F=3, M=3, J=3, A=3
	Artis	N=4; 1280 min (320) min	F=2, M=1, J=1, A=2	F=3, M=1, J=1, A=3	
	Planckendael	N=5; 909 min (181.88±25.84) min			N=5, F=2, M=3, A=5

Table S2. Details of macaque study groups excluded from partial Bayesian correlation analyses. Justification of inclusion/exclusion of study groups in different statistical analyses are provided in the methods; also see Table S1 and Data S1-S4. These justifications included – (i) group not tested due to institutional regulations for COVID-19, (ii) presence of all-male group, (iii) macaques not meeting training /self-participation criteria, and (iv) limited interactions among group members to calculate relevant measures, such as hierarchy steepness.

Partial Bayesian correlation	Study groups excluded (in order of appearance in results)
Within-group cooperation success and tolerance grades	4 groups: *M. mulatta-R3G7, M. silenus-Apenheul, M. sylvanus-Apenheul, M. nigra-Planckendael*
Tolerance grades and hierarchy steepness based on all group members	1 group: *M. fuscata-Affenberg*
Tolerance grades and hierarchy steepness based on only adult females	3 groups: *M. fuscata-Affenberg*, *M. fascicularis-NWR, M. silenus-Blijdorp*
Co-feeding tolerance and Pielou's *J'*	6 groups: *M. fuscata-Affenberg, M. mulatta-R3G2, M. silenus-Apenheul, M. sylvanus-Apenheul, M. nigra-Planckendael, M. nigra-Artis*
Within-group cooperation and co-feeding tolerance	6 groups: *M. fuscata-Affenberg, M. mulatta-R3G7, M. silenus-Apenheul, M. sylvanus-Apenheul, M. nigra-Planckendael, M. nigra-Artis*
Within-group cooperation and number of prosocial individuals	5 groups: *M. mulatta-R3G7, M. mulatta-R3G2, M. silenus-Apenheul, M. sylvanus-Apenheul, M. nigra-Planckendael*
Grooming transitivity, reciprocity, and modularity and tolerance grades	1 group: *M. fuscata-Affenberg*

Table S3. Effects of dyadic tolerance, rank difference, tolerance grades, prosociality, and kinship on the likelihood of cooperation.

Family: bernoulli
Links: mu = logit

Formula: likelihood_coop ~ 0 + Intercept + dyadic tolerance + rank difference + grade + pros ocial + kinship + (1 | group:ind1) + (1 | group:ind2) + (1 | species/group)

Data: data (Number of observations: 214)

Draws: 4 chains, each with iter = 5500; warmup = 1000; thin = 1; post-warmup draws = 1800 0

	Estimate	89% critical interval	Probability of direction
Dyadic tolerance	0.86	0.40, 1.37	0.99
Rank difference	-0.37	-0.83, 0.07	0.91
Grade [1]	-0.28	-1.64, 1.10	0.63
Grade [2]	-0.12	-1.45, 1.23	0.55
Grade [3]	-0.28	-1.69, 1.14	0.62
Prosocial [Yes]	1.41	0.58, 2.27	0.99
Kinship [Yes]	1.23	0.29, 2.18	0.98

Note: only best-fitted model is reported.

Table S4. Effects of dyadic tolerance, rank difference, tolerance grades, prosociality, and kinship on the magnitude of cooperation.

Family: negbinomial
Links: mu = log; shape = identity
Formula: magnitude_coop ~ 0 + Intercept + dyadic tolerance + rank difference + grade + pro social + kinship + offset(log(trials)) + (1 | group:ind1) + (1 | group:ind2) + (1 | species/group)
Data: data_m (Number of observations: 80)
Draws: 4 chains, each with iter = 5500; warmup = 1000; thin = 1; post-warmup draws = 1800 0

	Estimate	89% critical interval	Probability of direction
Dyadic tolerance	0.23	0.09, 0.40	0.99
Rank difference	-0.17	-0.46, 0.11	0.83
Grade [1]	-0.51	-1.68, 0.72	0.76
Grade [2]	0.24	-0.96, 1.35	0.64
Grade [3]	0.37	-0.85, 1.56	0.69
Prosocial [Yes]	0.46	-0.11, 1.03	0.9
Kinship [Yes]	-0.45	-1.03, 0.13	0.89

Note: only best-fitted model is reported.

Table S5. Effects of tolerance grades, group size, groom index, and aggression index on the likelihood of cooperation.

Family: bernoulli
Links: mu = logit
Formula: likelihood_coop ~ 0 + Intercept + grade + group size + groom index + aggression in dex + (1 | group:ind1) + (1 | group:ind2) + (1 | species/group)
Data: data (Number of observations: 115)
Draws: 4 chains, each with iter = 5500; warmup = 1000; thin = 1; post-warmup draws = 1800 0

	**Estimate**	**89% critical interval**	**Probability of direction**
Grade [1]	-0.20	-1.64, 1.23	0.59
Grade [2]	0.11	-1.21, 1.44	0.55
Grade [3]	-0.24	-1.60, 1.12	0.61
Group size	-0.26	-0.48, -0.07	0.98
Groom index	0.17	-0.30, 0.63	0.73
Aggression index	0.47	-0.00, 0.96	0.94

Note: only best-fitted model is reported.

Table S6. Effects of tolerance grades, group size, groom index, and aggression index on the magnitude of cooperation.

Family: negbinomial
Links: mu = log; shape = identity

Formula: magnitude_coop ~ 0 + Intercept + grade + group size + groom index + aggression i ndex + offset(log(trials)) + (1 | group:ind1) + (1 | group:ind2) + (1 | species/group)

Data: data (Number of observations: 47)

Draws: 4 chains, each with iter = 5000; warmup = 1000; thin = 1; post-warmup draws = 1600 0

	Estimate	89% critical interval	Probability of direction
Grade [1]	0.27	-1.07, 1.60	0.63
Grade [2]	0.27	-0.98, 1.44	0.65
Grade [3]	0.18	-1.08, 1.41	0.59
Group size	-0.07	-0.19, 0.04	0.84
Groom index	-0.11	-0.42, 0.23	0.70
Aggression index	0.26	-0.07, 0.60	0.90

Note: only best-fitted model is reported.

Table S7. Effects of dyadic tolerance, rank difference, tolerance grades, and kinship on the likelihood of prosocial food provisioning.

Family: bernoulli
Links: mu = logit

Formula: provision_likelihood ~ 0 + Intercept + dyadic tolerance + rank difference + grade + kinship + (1 | group/ind1/ind2) + (1 | species/group)

Data: data (Number of observations: 302)

Draws: 4 chains, each with iter = 5500; warmup = 1000; thin = 1; post-warmup draws = 1800 0

	**Estimate**	**89% critical interval**	**Probability of direction**
Dyadic tolerance	0.98	0.57, 1.53	1.00
Rank difference	-0.28	-0.76, 0.14	0.85
Grade [1]	-0.56	-1.93, 0.86	0.74
Grade [2]	0.21	-1.18, 1.59	0.59
Grade [3]	0.35	-1.16, 1.82	0.65
Kinship [Yes]	1.13	0.33, 1.96	0.98

Note: only best-fitted model is reported.

Table S8. Effects of dyadic tolerance, rank difference, and kinship on the magnitude of prosocial food provisioning.

Family: negbinomial
Links: mu = log; shape = identity

Formula: Provision_magnitude ~ 0 + Intercept + dyadic tolerance + rank difference + kinship + offset(log(trials)) + (1 | group/ind1/ind2) + (1 | species/group)

Data: data (Number of observations: 49)

Draws: 4 chains, each with iter = 5500; warmup = 1000; thin = 1; post-warmup draws = 1800 0

	Estimate	89% critical interval	Probability of direction
Dyadic tolerance	0.10	-0.02, 0.22	0.90
Rank difference	-0.09	-0.46, 0.28	0.64
Kinship [Yes]	0.98	0.45, 1.51	0.99

Note: only best-fitted model is reported.

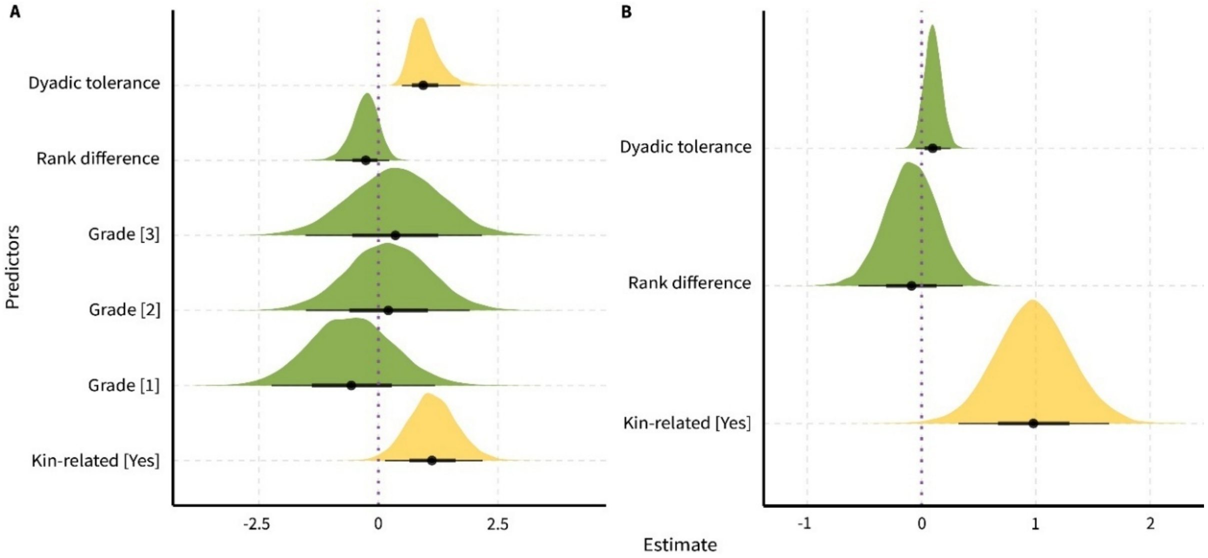

Fig. S1. Dyadic predictors of prosocial food provisioning. (A) Posterior effects of dyadic social tolerance, rank difference, tolerance grades, and kinship on the likelihood of prosocial food provisioning, estimated from a Bayesian mixed-effects model (n = 302 dyads). Only strong effects of dyadic social tolerance (Est = 0.98, 89% crI = [0.57, 1.53]) and kin-relation (Est = 1.13, 89% crI = [0.33, 1.96]) were found. **(B)** Posterior effects of dyadic social tolerance, rank difference, and kinship on the magnitude of prosocial food provisioning, estimated from a Bayesian mixed-effects model (n = 49 dyads). Only strong effect of kin relation was found (Est = 0.98, 89% crI = [0.45, 1.51]). Yellow colors indicate strong effects; width of 'half-eye' represents data distribution (89% crI); solid black points on horizontal bars indicate median values; vertical purple dashed lines indicate a parameter estimate of zero, i.e., overlap of the crI with this line suggests no effects. Source data are provided as a Source Data file.

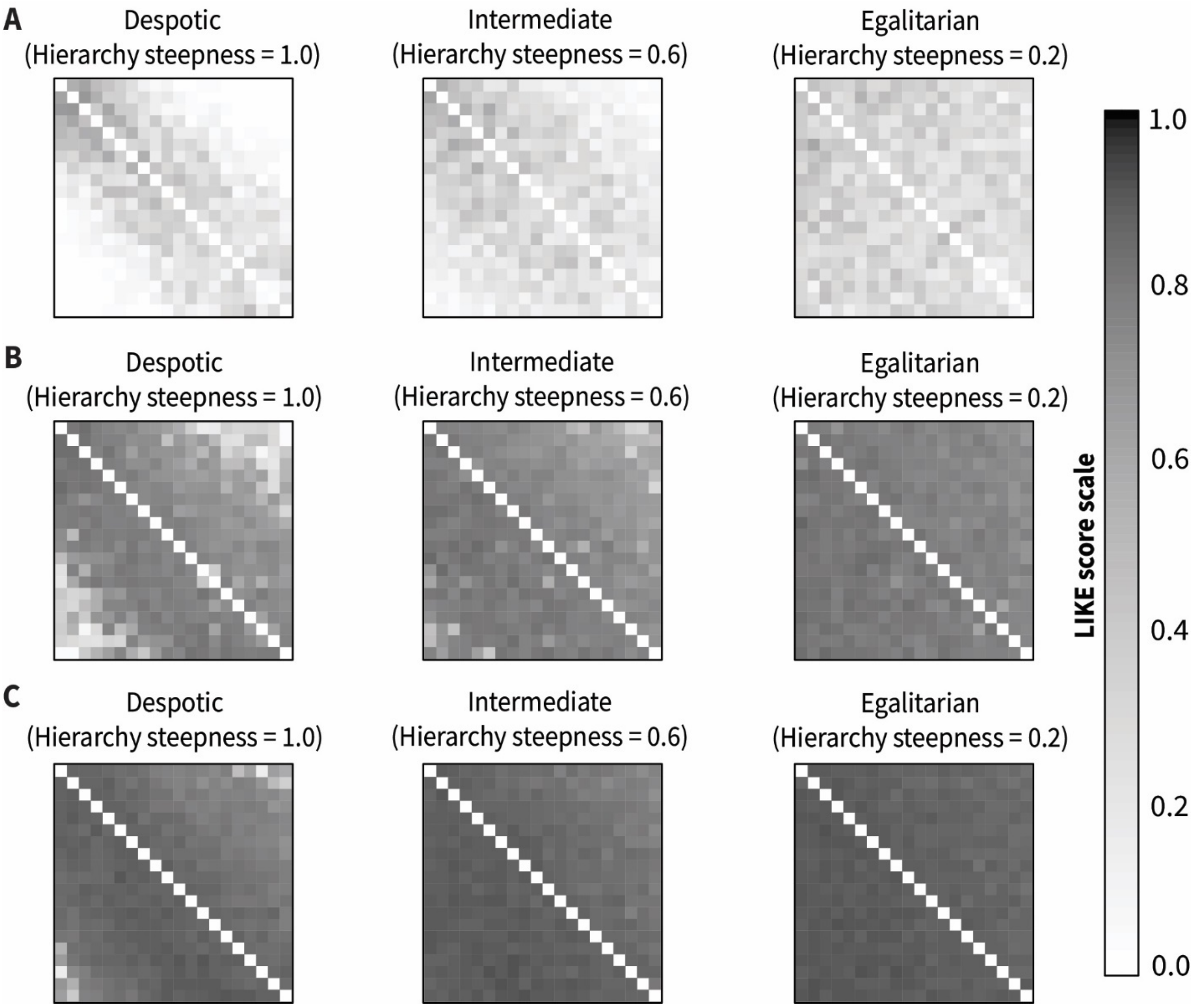

Fig. S2. EMO-model simulation with easy-going LIKE dynamics and fast increase speed shows the emergence of LIKE relationships in societies along a despotic-egalitarian gradient. (A) LIKE relationships in societies with a fast decrease speed. **(B)** LIKE relationships in societies with an intermediate decrease speed. **(C)** LIKE relationships in societies with a slow decrease speed. On the y-axes, individuals are ordered from low ranking (top row) to high ranking (bottom row), and on the x-axes, from low ranking (left) to high ranking (right). Each square represents a LIKE attitude from one individual to another, indicating their LIKE relationship. A LIKE relationship ranges from 0.01 (white) to 0.99 (black), with higher values indicating stronger bonds. Source data are provided as a Source Data file.

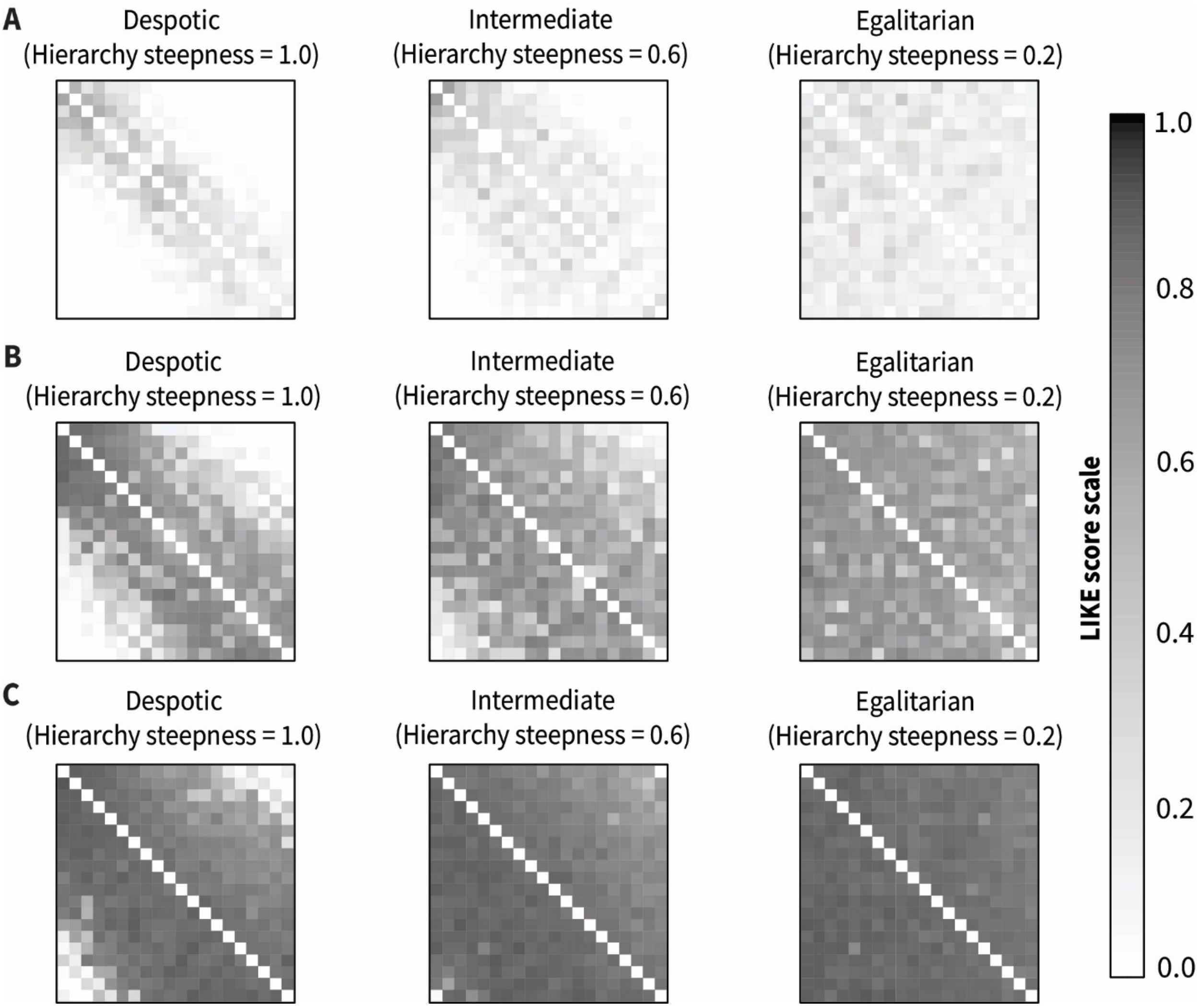

Fig. S3. EMO-model simulation with easy-going LIKE dynamics and intermediate increase speed shows the emergence of LIKE relationships in societies along a despotic-egalitarian gradient. (A) LIKE relationships in societies with a fast decrease speed. **(B)** LIKE relationships in societies with an intermediate decrease speed. **(C)** LIKE relationships in societies with a slow decrease speed. On the y-axes, individuals are ordered from low ranking (top row) to high ranking (bottom row), and on the x-axes, from low ranking (left) to high ranking (right). Each square represents a LIKE attitude from one individual to another, indicating their LIKE relationship. A LIKE relationship ranges from 0.01 (white) to 0.99 (black), with higher values indicating stronger bonds. Source data are provided as a Source Data file.

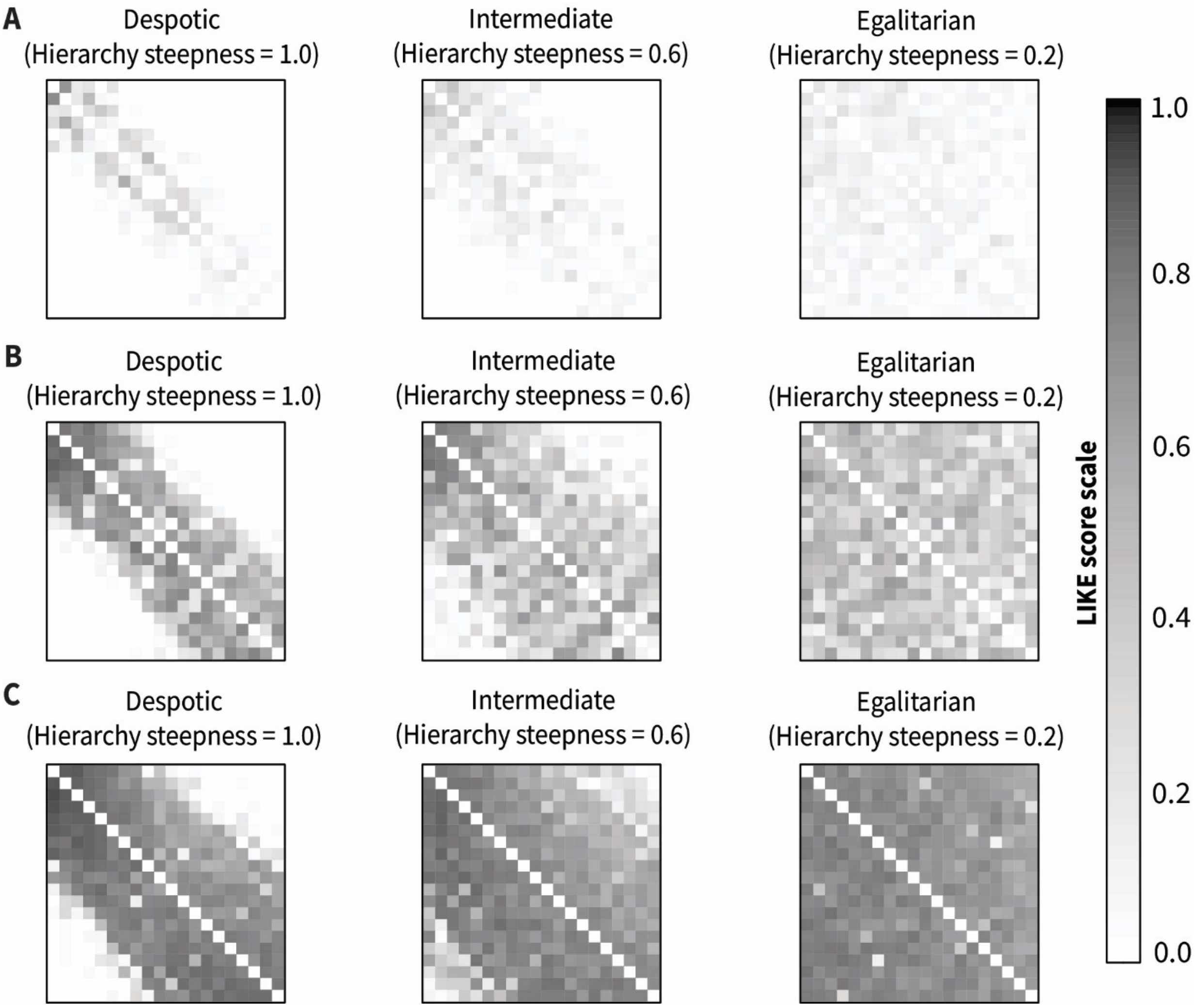

Fig. S4. EMO-model simulation with easy-going LIKE dynamics and slow increase speed shows the emergence of LIKE relationships in societies along a despotic-egalitarian gradient. (A) LIKE relationships in societies with a fast decrease speed. **(B)** LIKE relationships in societies with an intermediate decrease speed. **(C)** LIKE relationships in societies with a slow decrease speed. On the y-axes, individuals are ordered from low ranking (top row) to high ranking (bottom row), and on the x-axes, from low ranking (left) to high ranking (right). Each square represents a LIKE attitude from one individual to another, indicating their LIKE relationship. A LIKE relationship ranges from 0.01 (white) to 0.99 (black), with higher values indicating stronger bonds. Source data are provided as a Source Data file.

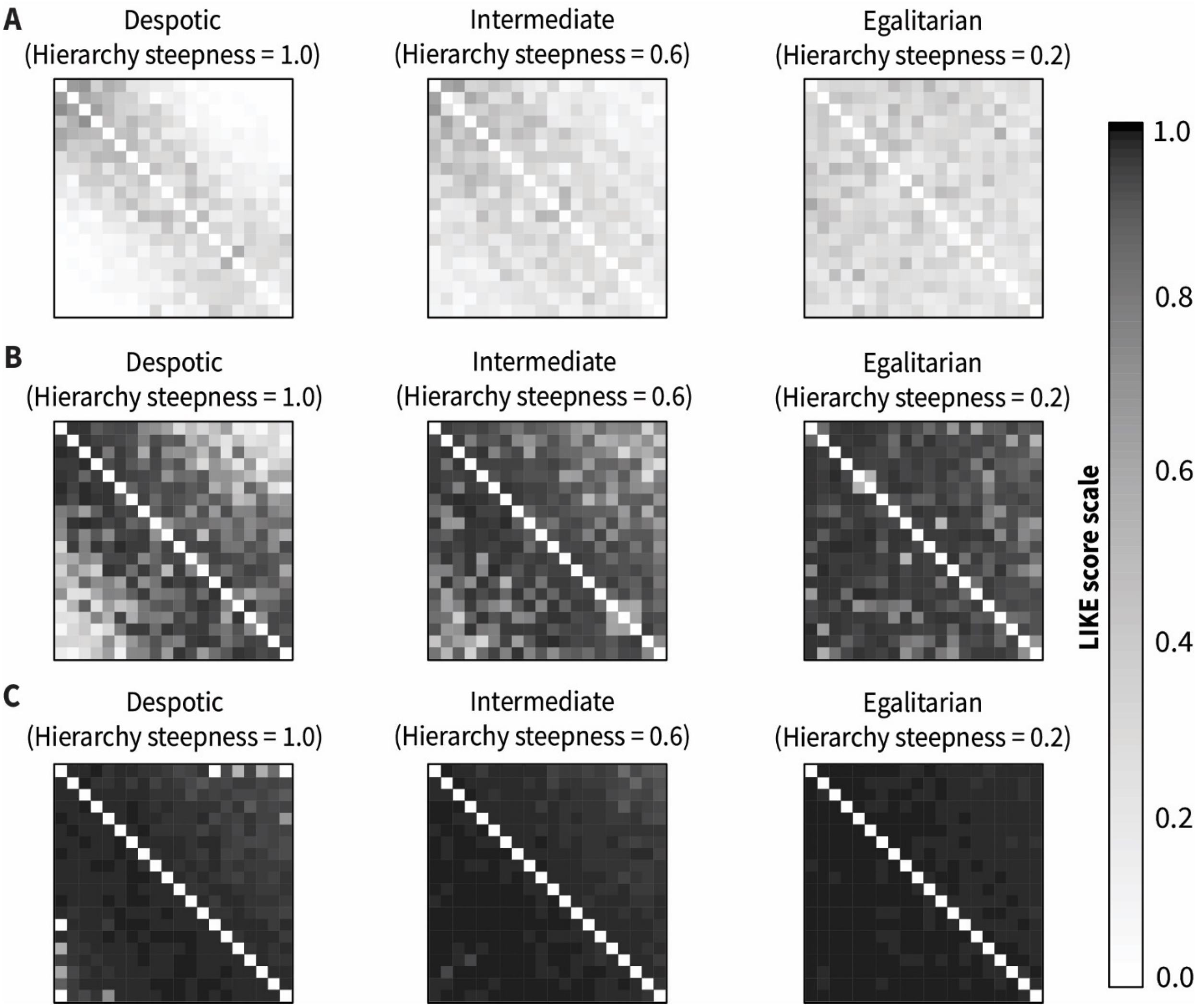

Fig. S5. EMO-model simulation with picky LIKE dynamics and fast increase speed shows the emergence of LIKE relationships in societies along a despotic-egalitarian gradient. (A) LIKE relationships in societies with a fast decrease speed. **(B)** LIKE relationships in societies with an intermediate decrease speed. **(C)** LIKE relationships in societies with a slow decrease speed. On the y-axes, individuals are ordered from low ranking (top row) to high ranking (bottom row), and on the x-axes, from low ranking (left) to high ranking (right). Each square represents a LIKE attitude from one individual to another, indicating their LIKE relationship. A LIKE relationship ranges from 0.01 (white) to 0.99 (black), with higher values indicating stronger bonds. Source data are provided as a Source Data file.

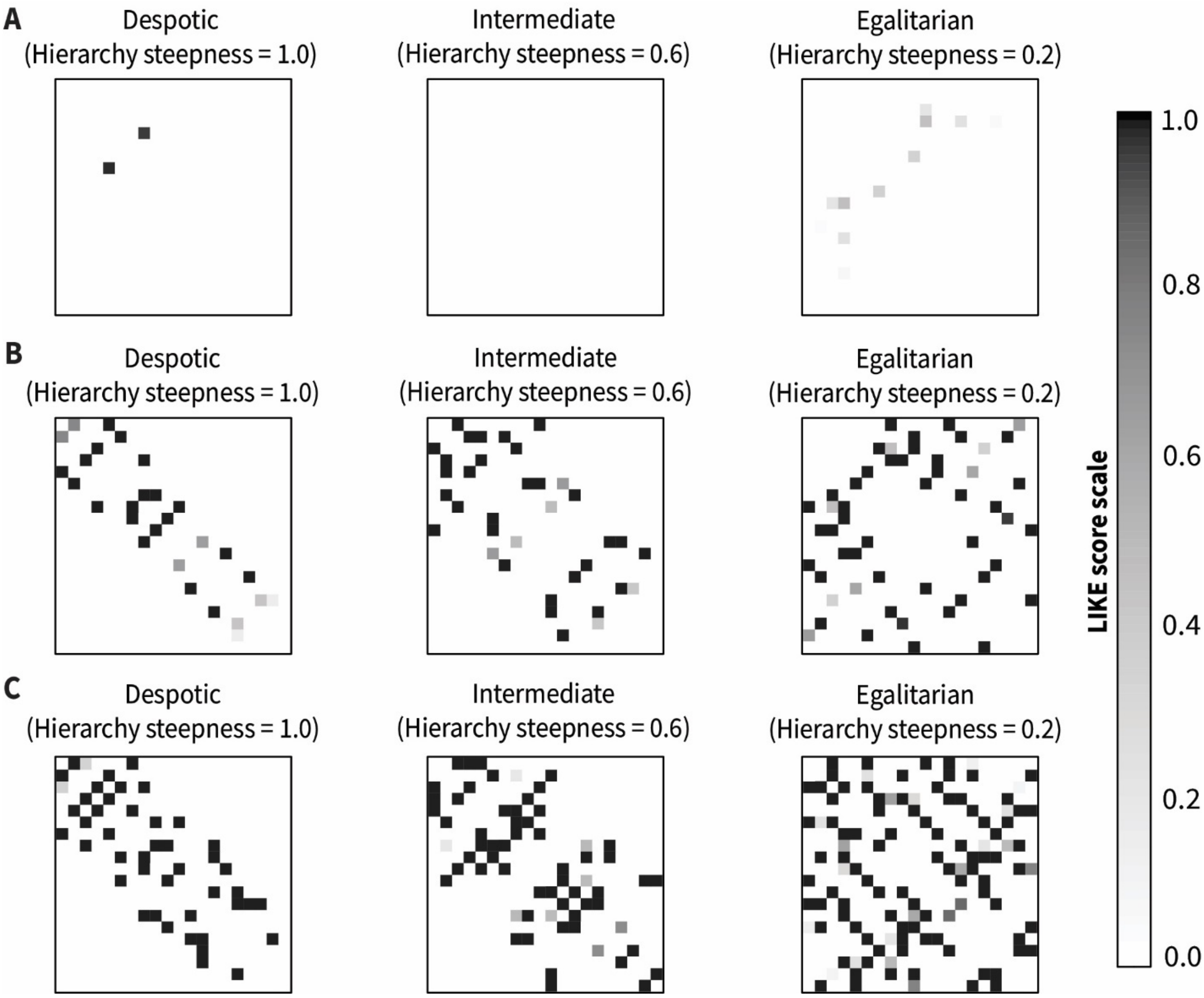

Fig. S6. EMO-model simulation with picky LIKE dynamics and slow increase speed shows the emergence of LIKE relationships in societies along a despotic-egalitarian gradient. (A) LIKE relationships in societies with a fast decrease speed. **(B)** LIKE relationships in societies with an intermediate decrease speed. **(C)** LIKE relationships in societies with a slow decrease speed. On the y-axes, individuals are ordered from low ranking (top row) to high ranking (bottom row), and on the x-axes, from low ranking (left) to high ranking (right). Each square represents a LIKE attitude from one individual to another, indicating their LIKE relationship. A LIKE relationship ranges from 0.01 (white) to 0.99 (black), with higher values indicating stronger bonds. Source data are provided as a Source Data file.

References

1. Bhattacharjee, D., Cousin, E., Pflüger, L. S. & Massen, J. J. M. Prosociality in a despotic society. *iScience* **26**, 106587 (2023).
2. Bhattacharjee, D., Waasdorp, S., Middelburg, E., Sterck, E. H. M. & Massen, J. J. M. Personality heterophily and friendship as drivers for successful cooperation. *Proceedings of the Royal Society B: Biological Sciences* **291**, (2024).
3. de Laat, E. J. A. M., Waasdorp, S., Roth, T. S., Massen, J. J. M. & Sterck, E. H. M. Despotic long-tailed macaques benefit others in a group service paradigm. *Behaviour* 1–29 (2025).
