# Supplementary material for: Despotism promotes dyadic cooperation through enhanced interdependencies in non-human primate societies
Source: Nat Commun. 2026 Apr 30;17:3513. doi: 10.1038/s41467-026-71168-7 (PMC13133276; doi:10.1038/s41467-026-71168-7)
Supplement: Supplementary file 2 — Description of Additional Supplementary Files [file 41467_2026_71168_MOESM2_ESM.pdf]

Description of Additional Supplementary Files

File name: Supplementary Movie 1

Description: Testing phase of the loose-string paradigm for cooperation in *Macaca mulatta*. Two macaques simultaneously pulled the two loose ends of a single string and obtained food rewards.

File name: Supplementary Movie 2

Description: Test condition of the prosocial group service paradigm in *Macaca silenus*. An individual pressed the seesaw handle and provisioned food reward to another individual.

File name: Supplementary Movie 3

Description: Food distribution assessment phase of the prosocial group service paradigm in *Macaca mulatta*.

File name: Supplementary Movie 4

Description: Co-feeding peanut plot experiment for quantifying within-group social tolerance in *Macaca silenus*.

File name: Supplementary Data 1

Description: Details of individuals qualifying the loose-string cooperation paradigm criteria.

File name: Supplementary Data 2

Description: Details of individuals with proactive prosocial motivations based on the group service paradigm.

File name: Supplementary Data 3

Description: Number of individuals present within the co-feeding peanut plot over time.

File name: Supplementary Data 4

Description: Group-level details of cooperation and social interactions.

File name: Supplementary Data 5

Description: Number of cooperating and non-cooperating dyads belonging to the four macaque tolerance grades and the distribution of cooperation magnitude in cooperating dyads.
