# Supplementary material for: Despotism promotes dyadic cooperation through enhanced interdependencies in non-human primate societies
Source: Nat Commun. 2026 Apr 30;17:3513. doi: 10.1038/s41467-026-71168-7 (PMC13133276; doi:10.1038/s41467-026-71168-7)
Supplement: Supplementary file 12 — Reporting Summary [file 41467_2026_71168_MOESM12_ESM.pdf]

nature portfolio

Corresponding author(s): Debottam Bhattacharjee, Edwin JC van Leeuwen, Jorg JM Massen

Last updated by author(s): Jan 27, 2026

Reporting Summary

Nature Portfolio wishes to improve the reproducibility of the work that we publish. This form provides structure for consistency and transparency in reporting. For further information on Nature Portfolio policies, see our Editorial Policies and the Editorial Policy Checklist.

Statistics

For all statistical analyses, confirm that the following items are present in the figure legend, table legend, main text, or Methods section.

n/a	Confirmed	
☐	☒	The exact sample size (*n*) for each experimental group/condition, given as a discrete number and unit of measurement
☐	☒	A statement on whether measurements were taken from distinct samples or whether the same sample was measured repeatedly
☐	☒	The statistical test(s) used AND whether they are one- or two-sided<br>*Only common tests should be described solely by name; describe more complex techniques in the Methods section.*
☐	☒	A description of all covariates tested
☐	☒	A description of any assumptions or corrections, such as tests of normality and adjustment for multiple comparisons
☐	☒	A full description of the statistical parameters including central tendency (e.g. means) or other basic estimates (e.g. regression coefficient) AND variation (e.g. standard deviation) or associated estimates of uncertainty (e.g. confidence intervals)
☐	☒	For null hypothesis testing, the test statistic (e.g. *F*, *t*, *r*) with confidence intervals, effect sizes, degrees of freedom and *P* value noted<br>*Give P values as exact values whenever suitable.*
☐	☒	For Bayesian analysis, information on the choice of priors and Markov chain Monte Carlo settings
☐	☒	For hierarchical and complex designs, identification of the appropriate level for tests and full reporting of outcomes
☐	☒	Estimates of effect sizes (e.g. Cohen's *d*, Pearson's *r*), indicating how they were calculated

Our web collection on statistics for biologists contains articles on many of the points above.

Software and code

Policy information about availability of computer code

Data collection	No software were used to collect data.
Data analysis	All data were coded from the video recordings using BORIS (version 7.13.6) or in a frame-by-frame manner using Pot player (version 240618). Statistical analyses were performed using R (version 4.4.1). The agent-based EMO-model was constructed using NetLogo (version 5.3.1).

For manuscripts utilizing custom algorithms or software that are central to the research but not yet described in published literature, software must be made available to editors and reviewers. We strongly encourage code deposition in a community repository (e.g. GitHub). See the Nature Portfolio guidelines for submitting code & software for further information.

Data

Policy information about availability of data

All manuscripts must include a data availability statement. This statement should provide the following information, where applicable:

- Accession codes, unique identifiers, or web links for publicly available datasets
- A description of any restrictions on data availability
- For clinical datasets or third party data, please ensure that the statement adheres to our policy

All data generated in this study are available within the article, its supplementary information, and on Zenodo https://doi.org/10.5281/zenodo.18366379. There are no restrictions on data availability. Source data are provided in this article. All code and accessory files to reproduce results are uploaded on Zenodo https://doi.org/10.5281/zenodo.18366379.

Research involving human participants, their data, or biological material

Policy information about studies with human participants or human data. See also policy information about sex, gender (identity/presentation), and sexual orientation and race, ethnicity and racism.

Reporting on sex and gender	We studied both male and female macaques in their existing social settings and without changing the existing social group compositions. They are all explicitly mentioned in the manuscript. We did not study human participants in this research. However, in relation to gender of co-authors in the research, they represent a diverse group of researchers from all genders (binary and non-binary).
Reporting on race, ethnicity, or other socially relevant groupings	We did not study human participants in this research. However, the co-authors represent a group of people from diverse geographic origins, ethnicities, and professional career stages.
Population characteristics	Not applicable.
Recruitment	Not applicable.
Ethics oversight	All observational and experimental study procedures were approved by the Animal Experiments Committee and Animal Welfare Organisation of Biomedical Primate Research Centre (Animal Welfare Organisation/IvD approval no.: 019A, 019C, 019D, and 019E). Furthermore, internal committees of all relevant zoos carefully monitored the study. All study components were non-invasive (European Directive 2010/63), and we strictly adhered to the ethical principles and guidelines of the American Society of Primatologists for the care and inclusion of animals. No animals were isolated from their existing social groups during our research.

Note that full information on the approval of the study protocol must also be provided in the manuscript.

Field-specific reporting

Please select the one below that is the best fit for your research. If you are not sure, read the appropriate sections before making your selection.

☐ Life sciences ☐ Behavioural & social sciences ☒ Ecological, evolutionary & environmental sciences

For a reference copy of the document with all sections, see nature.com/documents/nr-reporting-summary-flat.pdf

Ecological, evolutionary & environmental sciences study design

All studies must disclose on these points even when the disclosure is negative.

Study description	This is a multi-method non-invasive study of behavioral observations and experiments. We studied 13 captive groups of macaques belonging to six different species (Macaca fuscata, M. mulatta, M. fascicularis, M. silenus, M. sylvanus, and M. nigra) in their existing social settings.
Research sample	We studied 13 captive groups of macaques belonging to six different species (Macaca fuscata, M. mulatta, M. fascicularis, M. silenus, M. sylvanus, and M. nigra) in their existing social settings. Macaques in a comparative framework are ideal for testing the predictions of the key hypothesis of this research. Key details are provided in the introduction, methods, and statistics.
Sampling strategy	No sample size calculation was done before the experimentation. This was due to the nature of experiments where participation of animals is completely voluntary. Nonetheless, from the team's extensive expertise in animal science studies, particularly related to non-human primates, the reported sample size is much larger than most existing high-quality research in the field. We studied 10 groups and 102 individuals for cooperative loose-string experiment, 9 groups and 96 individuals for prosocial group-service experiment, 11 groups and 105 individuals for co-feeding tolerance experiment, and also carried out behavioral observations on 109 individuals 12 groups. Furthermore, we checked statistically in the Bayesian framework for effect sizes and data sufficiency by comparing observed and fitted models.
Data collection	Multiple experimenters were involved in the collection of data. However, the same experimenter(s) carried out all phases of a single test (cooperation, prosociality, and co-feeding tolerance) for a given study group to avoid potential experimenter bias. We used a randomized order in which behavioral observations, cooperation, and prosociality tests were conducted. The co-feeding tolerance test took place at the end for all study groups. Notably, the study groups were familiarized with the concerned experimenters and showed no distress during the investigation. See author contributions for experimenter identities.
Timing and spatial scale	Spatial scale is not relevant to research questions and data type collected for this work. The study began in November 2020 and ended in February 2023.
Data exclusions	We found extremely low participation in R3G7 (one of the M.mulatta groups). Notably, this is the same group in which we conducted additional training sessions. We therefore decided to discard the data on this group from cooperation analyses. Other data from this group was used in statistical analyses. It was not predetermined.
Reproducibility	We provided detailed methodologies for reproducibility of our various study components. All methodologies used are standardized and have been used in previous studies on other or similar animal species. Information on failed experimentation has also been provided and how it was dealt with in statistical analyses. Additionally, the R-script and README files provided minute details and every steps to reproduce the results.

Randomization	All experiments and observations followed a randomized order. Within group behavioral observations of individual animals, a pseudo-randomized order was used. The co-feeding tolerance test took place at the end for all study groups.
Blinding	Blinding was not possible in our experimental designs. However, it was relevant to the assignment of experimenters to study animal groups. Experimenters, at the time of data collection, had no concrete information about the response and predictor variables. However, a more nuanced approach was taken during the coding of data. We comprehensively checked for inter-rater reliability using intra-class correlation (ICC) tests among the trained coders. Overall agreement was high, with the ICC (3,k) ranging between 0.88 and 0.97.

Did the study involve field work? ☐ Yes ☒ No

Reporting for specific materials, systems and methods

We require information from authors about some types of materials, experimental systems and methods used in many studies. Here, indicate whether each material, system or method listed is relevant to your study. If you are not sure if a list item applies to your research, read the appropriate section before selecting a response.

Materials & experimental systems

n/a	Involved in the study
☒	☐ Antibodies
☒	☐ Eukaryotic cell lines
☒	☐ Palaeontology and archaeology
☐	☒ Animals and other organisms
☒	☐ Clinical data
☒	☐ Dual use research of concern
☒	☐ Plants

Methods

n/a	Involved in the study
☒	☐ ChIP-seq
☒	☐ Flow cytometry
☒	☐ MRI-based neuroimaging

Animals and other research organisms

Policy information about studies involving animals; ARRIVE guidelines recommended for reporting animal research, and Sex and Gender in Research

Laboratory animals	Six different macaque species: Macaca fuscata (Japanese), M. mulatta (Rhesus), M. fascicularis (Long-tailed), M. silenus (Lion-tailed), M. sylvanus (Barbary), and M. nigra (Crested). Please refer to the manuscript for their details.
Wild animals	Not applicable.
Reporting on sex	We studied both male and female macaques in their existing social settings and without changing the existing social group compositions. They are all explicitly mentioned in the manuscript.
Field-collected samples	The husbandry protocols differed slightly across study groups due to species-specific requirements and in-house management decisions, but these protocols adhered to the European Association of Zoos and Aquaria guidelines for accommodation and care for animals. Accordingly, all enclosures had multiple enrichment structures, like climbing platforms, hanging ropes, wooden structures, tree trunks, and slides. Except for the M. fuscata and Apenheul M. sylvanus groups, which live exclusively in outdoor enclosures, indoor enclosures of all groups were temperature-controlled and had concrete floors covered with sawdust bedding. Depending on the nutritional requirements, feeding routines also varied across groups. The diet primarily consisted of monkey pellets, fresh vegetables and fruits, and seed mix (e.g., sunflower and corn). All study groups had access to drinking water 24/7. No change in the regular feeding schedule was made for our study. The participation of individuals in all of our studies was completely voluntary.
Ethics oversight	All observational and experimental study procedures were approved by the Animal Experiments Committee and Animal Welfare Organisation of Biomedical Primate Research Centre (Animal Welfare Organisation/IvD approval no.: 019A, 019C, 019D, and 019E). Furthermore, internal committees of all relevant zoos carefully monitored the study. All study components were non-invasive (European Directive 2010/63), and we strictly adhered to the ethical principles and guidelines of the American Society of Primatologists for the care and inclusion of animals. No animals were isolated from their existing social groups during our research.

Note that full information on the approval of the study protocol must also be provided in the manuscript.

Plants

Seed stocks	Not applicable.
Novel plant genotypes	Not applicable.
Authentication	Not applicable.
